# Supplementary material for: Avoidance Behavior in Patients with Chronic Dizziness: A Prospective Observational Study
Source: J Clin Med. 2022 Dec 16;11(24):7473. doi: 10.3390/jcm11247473 (PMC9785738; doi:10.3390/jcm11247473)
Supplement: Supplementary file 1 [file jcm-11-07473-s001.zip › jcm-2085160-supplementary.pdf]

Supplementary Table S1. Descriptive statistics (baseline) in patients with follow-up and patients without.

		Patients without follow-up data		Patients with follow-up data		
**Categorical variables**		**n**	**%**	**n**	**%**	**p**
sex	Female	195	33.0	172	29.1	0.112
	Male	134	22.7	90	15.2	
diagnosis category	Somatic	179	30.1	128	21.5	0.235
	Non- somatic	154	25.9	134	22.5	
duration of symptoms	<6 months	44	7.7	35	6.1	0.953
	>6 months	278	48.3	218	37.9	
clinical diagnosis	BPPV	7	1.2	11	1.8	0.340
	BV	14	2.4	12	2.0	
	CV	17	2.9	12	2.0	
	MD	27	4.5	18	3.0	
	MultD	40	6.7	35	5.9	
	PPPD	154	25.9	134	22.5	
	VM	17	2.9	10	1.7	
	VN	46	7.7	19	3.2	
	VP	5	0.8	5	0.8	
	VS	6	1.0	6	1.0	
continuous dizziness	Yes	184	32.4	132	23.2	0.356
	No	137	24.1	115	20.2	
attack-like	Yes	173	32.5	157	29.5	0.953
	No	123	23.1	80	15.0	

	Patients without follow-up data		Patients with follow-up data		
**Metric variables**	**mean**	**SD**	**mean**	**SD**	**p**
HADS anxiety	7.12	4.13	7.36	3.84	0.370
HADS depression	6.40	3.95	6.25	3.84	0.595.
mean MI alone baseline	2.31	1.17	2.32	1.05	0.454

Abbreviations: BPPV, benign paroxysmal positional vertigo; BV, bilateral vestibulopathy; CV,central vertigo; HADS, Hospital Anxiety and Depression Scale; MI, Mobility Inventory; MD, Meniere's disease; MultD, multisensory deficit; SD, standard deviation; VM, vestibular migraine; VN, vestibular neuritis; VP, vestibular paroxysmia; VS, vestibular schwannoma.

Supplementary Table S2. Descriptive Statistics of the Mobility inventory Avoidance Alone scale items.

	median	mean	Standard deviation	Interquartile range
MI1 cinema and theatre	2.0	2.466	1.581	3.0
MI2 supermarket	1.0	1.912	1.172	2.0
MI3 classrooms	1.0	1.654	1.091	1.0
MI4 shopping malls	2.0	2.191	1.324	2.0
MI5 restaurants	2.0	2.122	1.377	2.0
MI6 museums	1.0	2.021	1.400	2.0
MI7 elevators	1.0	2.058	1.447	2.0
MI8 auditoriums/stadiums	2.0	2.466	1.620	3.0
MI9 Garages	1.0	1.930	1.355	1.0
MI10 high places	2.0	2.584	1.568	3.0
MI11 enclosed spaces	1.0	2.167	1.443	2.0
MI12 open spaces, outside	1.0	1.566	0.978	1.0
MI13 open spaces, inside	1.0	1.736	1.129	1.0
MI14 buses	1.0	1.934	1.381	1.5
MI15 trains	1.0	1.894	1.379	1.0
MI16 subways	1.0	2.040	1.475	2.0
MI17 airplanes	2.0	2.584	1.725	4.0
MI18 boats	2.0	2.475	1.658	3.0
MI19 cars (at any time)	1.0	1.902	1.299	1.0
MI20 cars (on expressways)	1.0	2.112	1.411	2.0
MI21 standing in line	2.0	2.421	1.364	3.0
MI22 crossing bridges	1.0	1.715	1.138	1.0
MI23 parties or social gatherings	2.0	2.403	1.365	2.0
MI24 walking on the street	1.0	1.816	1.108	1.0
MI25 staying home alone	1.0	1.383	0.795	0.0
MI26 being far away from home	2.0	2.115	1.317	2.0

Scale: 1 = never avoid, 2 = rarely avoid, 3 = avoid about half of the time, 4 = avoid most of the time, 5 = always avoid.
